# Advances in applied homeostatic modelling of the relationship between thyrotropin and free thyroxine

**DOI:** 10.1371/journal.pone.0187232

**Published:** 2017-11-20

**Authors:** Rudolf Hoermann, John Edward Maurice Midgley, Rolf Larisch, Johannes Wolfgang Christian Dietrich

**Affiliations:** 1 Department for Nuclear Medicine, Klinikum Lüdenscheid, Paulmannshöherstr, Lüdenscheid, Germany; 2 North Lakes Clinical, 20 Wheatley Avenue, Ilkley, United Kingdom; 3 Medical Department I, Endocrinology and Diabetology, Bergmannsheil University Hospitals, Ruhr University of Bochum, Bochum, Germany; 4 Ruhr Center for Rare Diseases (CeSER), Ruhr University of Bochum, Alexandrinenstr. 5, Bochum, Germany; 5 Ruhr Center for Rare Diseases (CeSER), Witten/Herdecke University, Alexandrinenstr. 5, Bochum, Germany; UCLA, UNITED STATES

## Abstract

**Introduction:**

The relationship between pituitary TSH and thyroid hormones is central to our understanding of thyroid physiology and thyroid function testing. Here, we generated distribution patterns by using validated tools of thyroid modelling.

**Methods:**

We simulated patterns of individual set points under various conditions, based on a homeostatic model of thyroid feedback control. These were compared with observed data points derived from clinical trials.

**Results:**

A random mix of individual set points was reconstructed by simulative modelling with defined structural parameters. The pattern displayed by the cluster of hypothetical points resembled that observed in a natural control group. Moderate variation of the TSH-FT4 gradient over the functional range introduced further flexibility, implementing a scenario of adaptive set points. Such a scenario may be a realistic possibility for instance in treatment where relationships and equilibria between thyroid parameters are altered by various influences such as LT4 dose and conversion efficiency.

**Conclusions:**

We validated a physiologically based homeostatic model that permits simulative reconstruction of individual set points. This produced a pattern resembling the observed data under various conditions. Applied modelling, although still experimental at this stage, shows a potential to aid our physiological understanding of the interplay between TSH and thyroid hormones. It should eventually benefit personalised clinical decision making.

## Introduction

The rationale behind thyroid function testing has developed by exploiting the physiological feedback control between the pituitary and thyroid gland. This closely relates pituitary thyrotropin (TSH) to circulating free thyroid hormone concentrations [1]. Advances in TSH assay technology have provided clinicians with a sensitive screening tool for thyroid disorders [2–4]. This has also encouraged the tacit assumption that TSH measurement represents a direct mirror image of free thyroxine (FT4) levels that always accurately reflects the perceived optimum concentrations of the latter hormone. Arising from this belief and recognising its increased sensitivity, TSH has often become a substitute for the measurement of free T4 (FT4) and free T3 (FT3). This has inhibited the use of the direct tests in many circumstances. Recently, this simplistic view has been challenged by a series of studies demonstrating a more complex interrelationship between the two parameters [5–9]. Homeostatic equilibria between TSH and thyroid hormones have also been shown to differ in levothyroxine (LT4) -treated patients, compared to untreated controls [10]. Conventional reference limits for TSH are based on univariate population-statistics. These have been found to be technically inadequate for a control parameter such as TSH and thus are less suitable for individual diagnostic decision making [11,12]. Understanding these issues demands a thorough revisitation of the physiological principles that underpin the basis of the appropriate use and suitable interpretation both of TSH measurements and those of thyroid hormones [13]. Importantly, new evidence suggests that the relationship between TSH and FT4 may not be invariably fixed either across the functional spectrum or among individuals, but is highly individual and situational [14–16].

Theoretical modelling permits a simulative examination and interpretation of more complex scenarios. Mathematical models of thyroid hormone homeostasis have been developed by our group and others [14,17–23]. Some of the models are closely related to set point theory. A patient specific set point represents the intersecting point between the respective thyroid and pituitary response curves (nullclines or null isoclines). In an individual at equilibrium, this corresponds to a pair of measured FT4 and TSH values obtained at that time [14]. As an advantage over the widely used statistical consideration of independent univariate parameters this approach delivers a unified homeostatic measure provided that measurements are taken under steady state conditions [14,20–23]. Important differences between the two approaches become apparent when using a cluster of set points from healthy individuals in order to draw a reference area that includes 95% of such subjects. Whereas the independent single reference intervals for FT4 and TSH describe a rectangle, the set point distribution produces limits delineated by a kite-shaped area [14].

In this study, we applied physiologically based homeostatic modelling to simulate set points for the TSH-FT4 relationship. We examined whether model assumptions and reported structural parameters are suitable to produce clusters of hypothetical set points that closely match the observed pattern of real data points in health and disease [10,12]. Ultimately, reconstruction of set points could provide individual markers with less variation, compared to conventional reference ranges.

## Methods

### Modelling strategy

**Fig 1:**
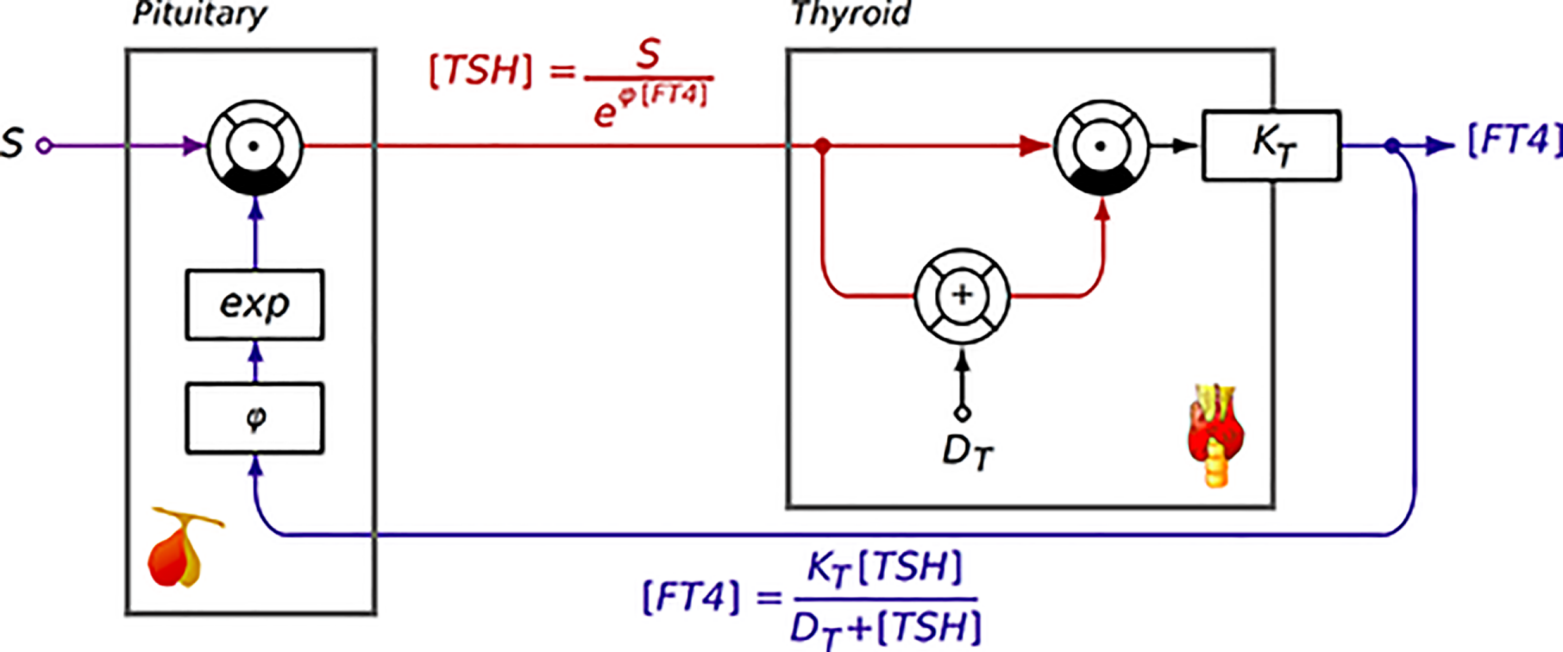
Information processing structure of the MiMe-Log model of thyroid homeostasis combining a Michaelis-Menten type feedforward path with logarithmic feedback inhibition. *S*: Set point, *phi*: slope of exponential coefficient, *K*_*T*_: Maximum T4 invasion, *D*_*T*_: EC50 of TSH at its receptor.

The general mathematical formalism is based on a hybrid model [20] combining a logarithmic description of feedback inhibition of TSH release by FT4 [20–27] and Michaelis-Menten kinetics in the feedforward path (Fig 1) [23–26,28]. Michealis-Menten kinetics lay the foundation for translational models bridging the gap between molecular biology and a systems-level approach. Related models can be studied either analytically [26] or with computer simulations [29]. Both approaches allow the mapping of physiological and biochemical parameters and their interplay involved in thyroid feedback control, as recently reviewed [14,23]. The logarithmic model of feedback inhibition is founded in the standard model of thyroid homeostasis [30]. Its advantage is its simple structure that allows for reconstructing the set point in open-loop situations as described below.

In closed-loop analysis the set point was conventionally defined as the intersecting point between the null isoclines of the feedback and feedforward path in the equilibrium state. The mathematical deconstruction of the two characteristic response curves of the pituitary and thyroid gland allowed for the identification of the set point [14]. Situated at the intersection of these two characteristic curves [14,27] the set point of an individual at a given time fulfils both the conditions of the feedforward and the feedback path. Therefore, it corresponds to a measured pair of TSH and FT4 values in a person under equilibrium conditions.

According to the MiMe-Log model [21] we used the following defining equations for the characteristic response curves of the pituitary or thyroid gland in our model [17,21,27].

1) The inverted pituitary response curve is defined by

$$[TSH]=Se^{–\varphi [FT4]},$$ 1.1.

where S refers to the intercept and phi to the gradient of the relationship. It may be rewritten as logarithmic expression

ln([TSH])=ln(S)–φ[FT4] 1.2.

in order to meet the formulation in earlier publications [30].

2) According to Michaelis-Menten kinetics the thyroid response curve is defined by:

$$[FT4]=\frac{K_{T}[TSH]}{D_{T}+[TSH]},$$ 2.1.

where *K*_*T*_ refers to maximum stimulated T4 invasion that results with

$$K_{T}=\frac{G_{T}\alpha_{T}}{\beta_{T}(1+K_{41}[TBG]+K_{42}[TTR])}$$ 2.2.

from the thyroid’s secretory capacity for T4 (*G*_*T*_) and parameters for distribution, elimination and plasma protein binding of T4 as previously described [14,17,23].

The set point was determined by the intersecting point from Eqs 1.2 and 2.1, which was efficiently solved with the use of the R package rootSolve 1.6.6 [31].

Set points in a hypothetical population were simulated by producing a cluster of individual set points with random variations of S and phi and deterministic changes in thyroid’s secretory capacity (*G*_*T*_). S and phi were randomly sampled from uniform or normal distributions, as described in Results.

In an observed population, the structural parameters S and phi were estimated from pairs of repeated TSH and FT4 measurements [20,21,27,32] with

$$\varphi =\frac{1}{{\left[FT4\right]}_{1}-{\left[FT4\right]}_{2}}ln(\frac{{\left[TSH\right]}_{2}}{{\left[TSH\right]}_{1}}),$$ 3.1.

and

$$S={\left[TSH\right]}_{1}e^{\varphi {\left[FT4\right]}_{1}}$$ 3.2.

The subscripts 1 or 2 refer to time points 1 or 2.

### Clinical data and laboratory methods

#### Patients

Simulations were compared to naturally observed data from recent clinical studies including a large prospective sample of consecutively seen patients [10] and a retrospective cohort of patients with differentiated thyroid carcinoma followed long-term after thyroidectomy and radioiodine treatment [33].

The protocol was approved by the Ethics Committee of the University of Muenster, Germany, and all participants gave their written informed consent. The trial has been registered at www.ClinicalTrials.gov (NCT 01969552, Study ID IIFHT-161013). Further details on patients and methods have been reported in previous articles [10,12,16,33,34].

We re-used a control group of euthyroid subjects (n = 268, 201 women, 67 men, mean (sd) age 47 (17) years), and a group of out-patients treated with LT4 for benign thyroid diseases (n = 170, 151 women, 23 men, age 55 (14) years, 83 with autoimmune thyroiditis, 87 with benign nodular goitre after surgery). For better comparability of these groups, patients with thyroid carcinoma were excluded and ranges restricted for FT4 (10–20 pmol/L) and TSH (0.1–4.0 mIU/L).

Of the thyroid carcinoma patients followed [33], we selected a third, longitudinal study group (n = 64, 39 women, 25 men, age 49 (16) years), based on two conditions that a change in LT4 dose ≥ 50 μg/day had occurred during follow-up and that TSH concentrations remained within an accurately measurable range of 0.1 to 4.0 mIU/L. This excludes patients with both lesser alterations between the two measurements and complete TSH suppression where phi cannot be reliably estimated. From duplicate measurements corresponding to the maximum and minimum TSH and FT4 values phi was estimated for each individual patient, as described above. Phi values were then correlated with averaged FT4 concentrations to determine whether they followed a random pattern or were dependent on thyroid function.

#### Thyroid parameters

Third-generation TSH and free thyroid hormones were measured on the automated Siemens ADVIA Centaur XP platform (Siemens Healthcare Diagnostics, Erlangen, Germany). Reference intervals were as follows, 0.4 to 4.0 mIU/L for TSH, 3.1 to 6.8 pmol/L for FT3, 10 to 23 pmol/L for FT4 [35].

SPINA-GD (${\hat{G}}_{D}$, nmol/s) estimates the global activity of peripheral step-up deiodinases (i.e. the sum of type 1 and type 2 deiodinase activity controlling conversion of T4 to T3) from equilibrium levels of FT3, FT4 and estimated constants for plasma protein binding, distribution and elimination with [14,23,25]

$${\hat{G}}_{D}=\frac{\beta_{31}(K_{M1}+[FT4])(1+K_{30}[TBG])[FT3]}{\alpha_{31}[FT4]}.$$ 4.1.

### Statistical methods

Statistical correlations were based on Kendall’s tau rank correlation and multivariable linear models (GLM). The latter were statistically compared by ANOVA. Non-parametric testing and linear approximation were used for comparison of the clinical groups, as this was sufficient for the restricted range, and the more complex non-linear modelling equations failed to converge when limiting the data range. A two-sided p-value below 0.05 was considered significant. All calculations were performed on the statistical software platform R 3.3.1 for Mac [36].

## Results

**Fig 2:**
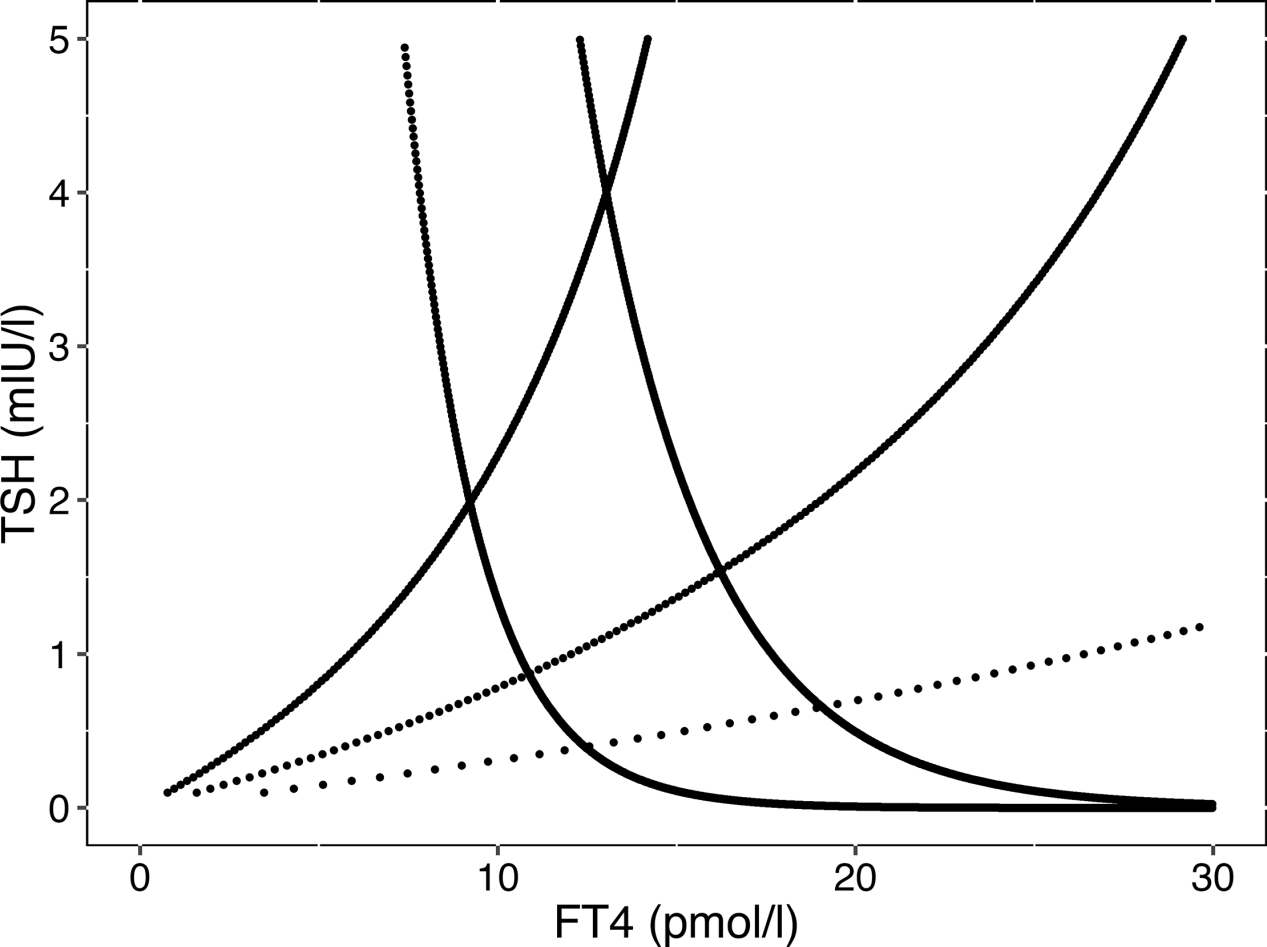
Reconstruction of set points. The illustration shows three thyroid response curves (TR1, TR2, TR3) and two inverted pituitary response curves (PR1, PR2). The thyroid curves were derived by a homeostatic model (see Methods) using different levels of maximum thyroid capacity (*G*_*T*_ = 1.67, 3.43 or 7.51 pmol/s), which correspond to the average and 95% confidence intervals from a previous study [16]. The pituitary response curves share the same maximum pituitary response (intercept S = 200), but vary in their gradient (phi = 0.3 or 0.5). The intersections of the curves define six individual set points.

According to set point theory, the measured pair of an FT4 value and a corresponding TSH level at equilibrium lies at the intersection of the thyroid and inverted pituitary response curves, as illustrated by Fig 2.

**Fig 3:**
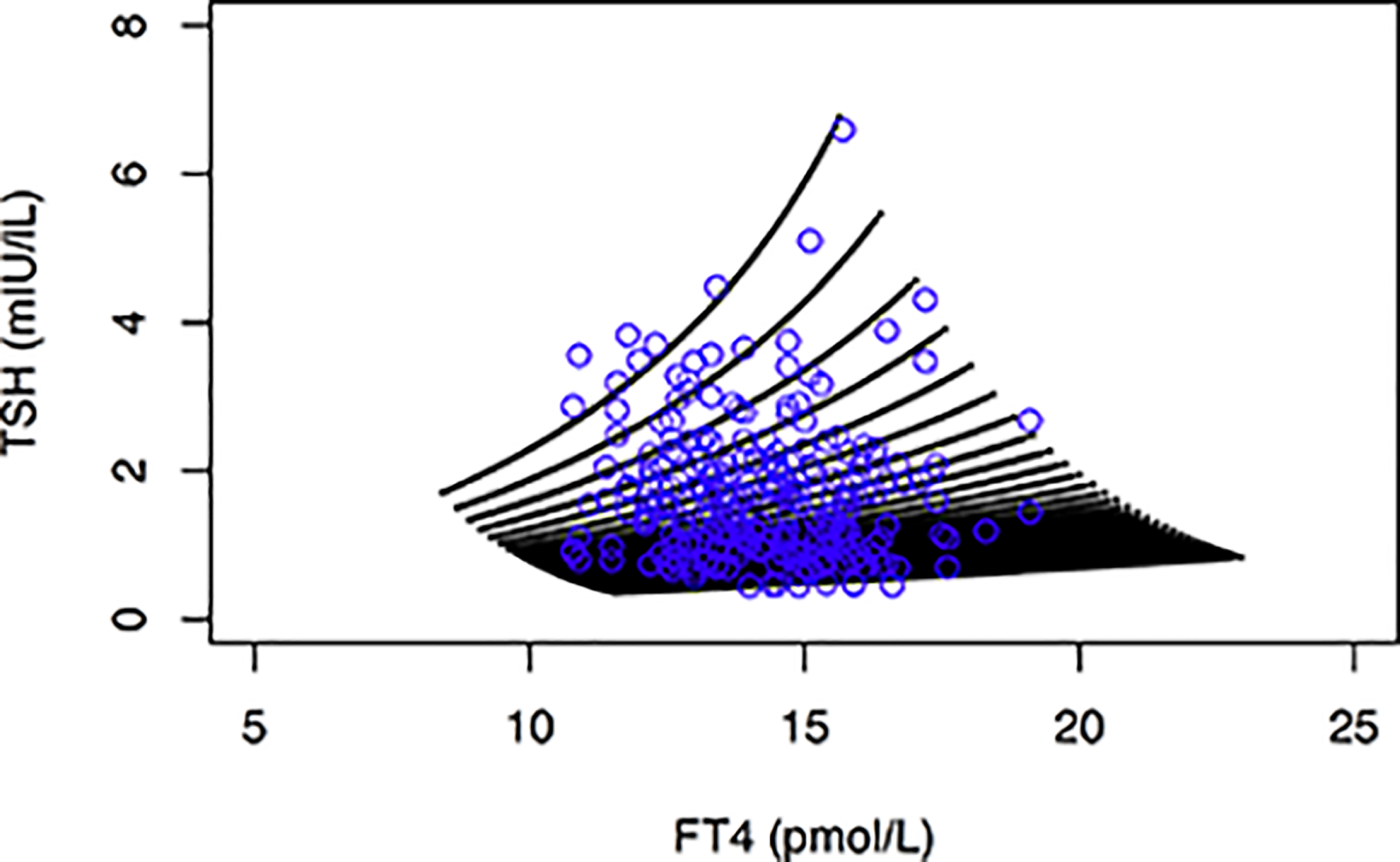
Cluster of random individual set points (black symbols) created by simulation with a homeostatic model. For comparison, the observed data points from a euthyroid control group (blue symbols) were overlaid. The thyroid response curve was constructed using a *G*_*T*_ sequence from 1.67 to 7.51 pmol/s (95% CI of the observed group) with an interval of 0.2. For constructing various individual pituitary response curves both S and phi values of 50 each were randomly sampled from uniform distributions ranging from 100 to 600 and 0.27 to 0.50, respectively. The resulting 75,000 intersecting points represent the clustered set points, as shown in the figure. For set point reconstruction refer to Methods and see Fig 1. For details on the clinical control group refer to Methods.

Assuming that each individual has its own defining structural parameters such as a maximum thyroid capacity (*G*_*T*_), maximum pituitary capacity (*S*) and a fixed trajectory for the gradient of the TSH-FT4 relationship (*phi*), we can create a random mix of hypothetical set points (Fig 3). The individual points cover a kite-shaped area, as theoretically expected. This resembles closely the observed pattern in a healthy population (Fig 3).

**Fig 4:**
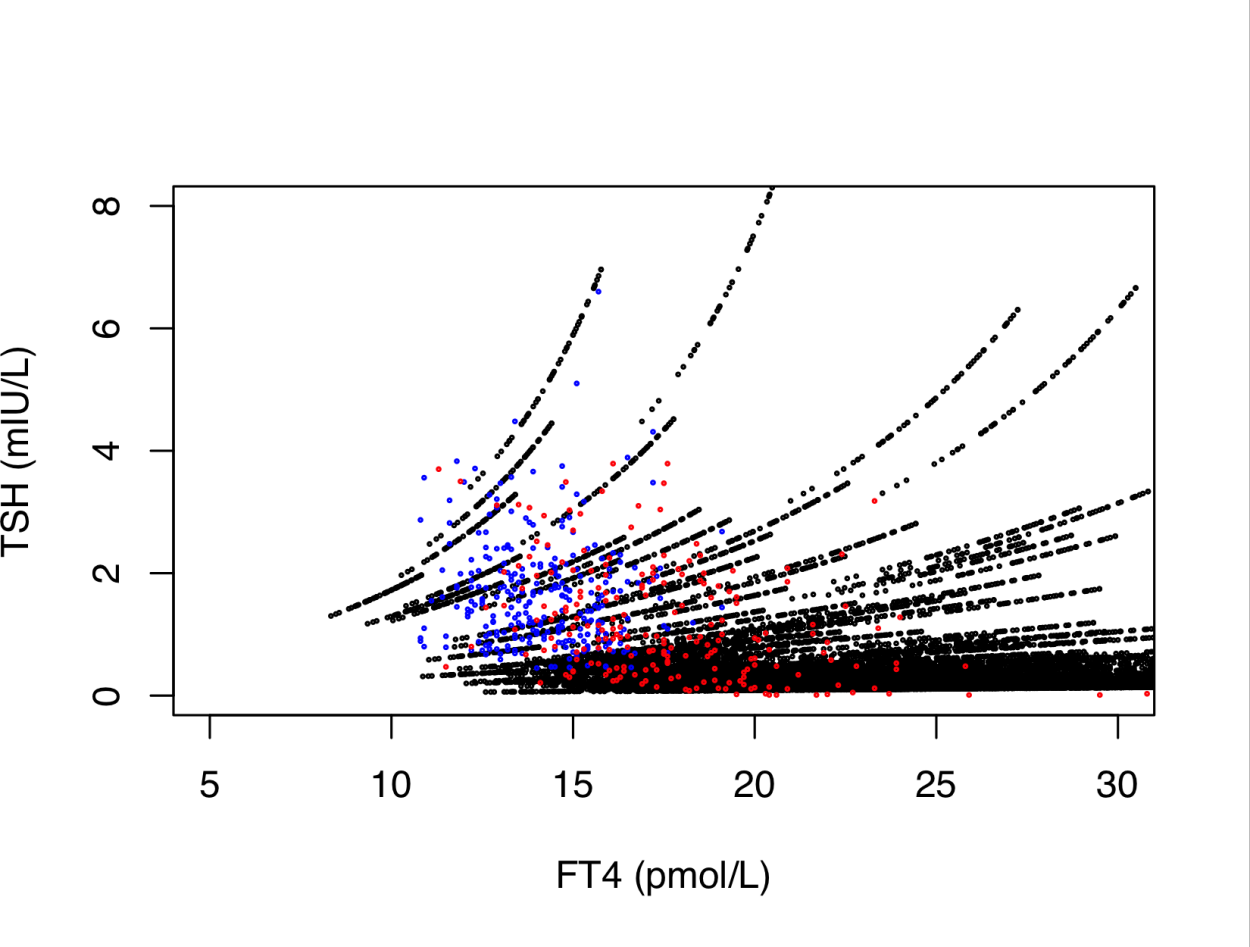
Phi-adjusted cluster of random individual set points (black symbols). The overlay depicts observed data points from euthyroid controls (n = 268, blue symbols) and LT4-treated patients (n = 207, red symbols). This simulates the possibility of a gradient change in the TSH-FT4 relationship. Reconstruction was done as described in Fig 2, but, unlike in Fig 2, the gradient phi was not fixed, rather continuously increased over the extended *G*_*T*_ range (1.67 to 50 nmol/s). It was randomly sampled from a normal distribution (mean 0.3, sd 0.08).

Introducing further flexibility to the model, we moderately varied the gradient (*phi*) or intercept (*S*) of the TSH-FT4 relationship dependent on *G*_*T*_ (Fig 4). The latter model involving adjustable set points in an individual person appeared to be suitable for describing variation observed in thyroid functional disease and a dose-dependent variation induced by LT4 treatment (Fig 4).

**Fig 5:**
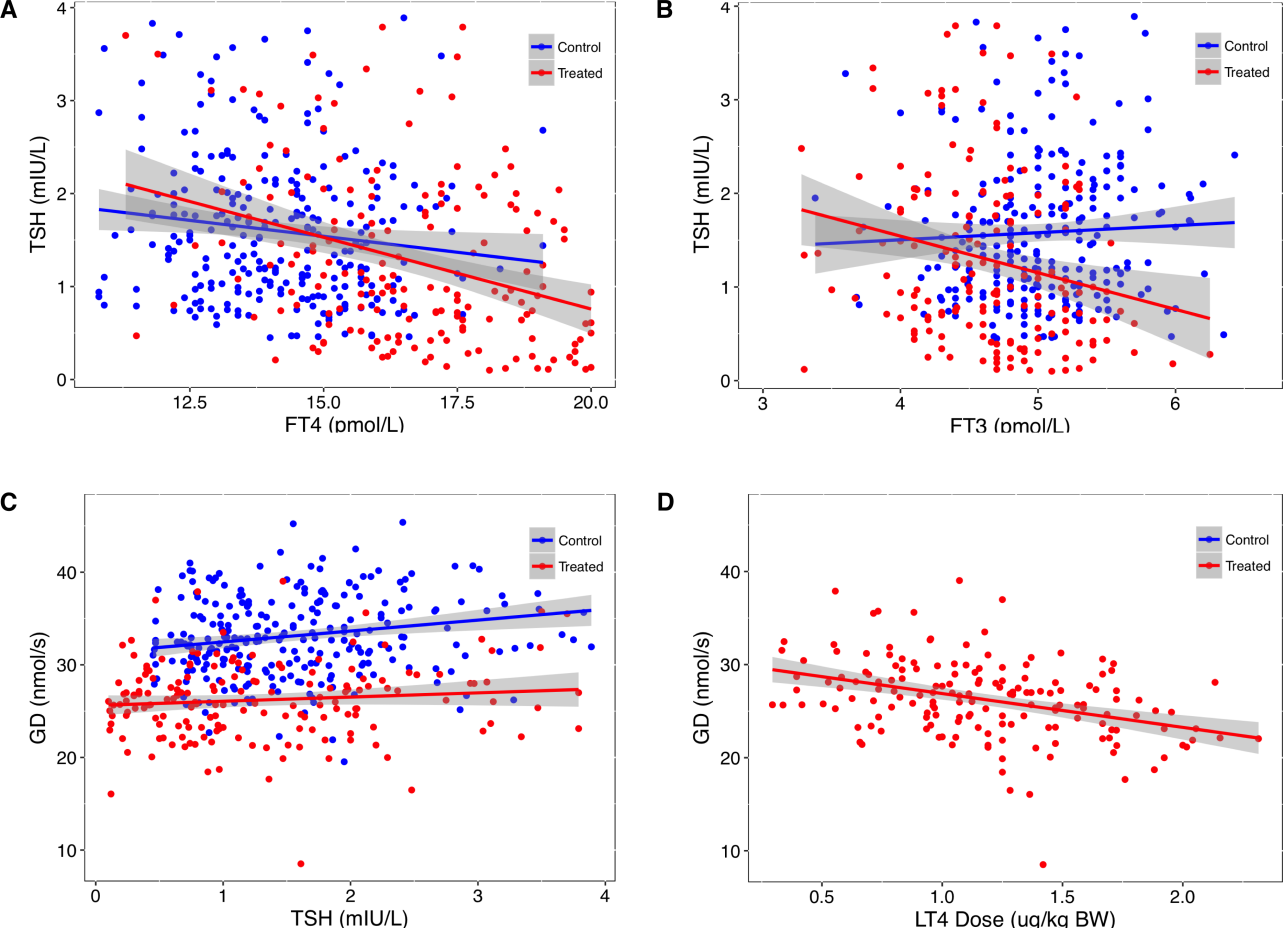
**Relationships between TSH and FT4 (A), FT3 (B) and step-up deiodinase activity (*G***_***D***_**, C) in a control group of healthy subjects and a group of LT4-treated patients with benign thyroid disease.** The correlation between *G*_*D*_ and weight adjusted L-T4 dose is shown in panel D. Regression lines are surrounded by the 95% confidence interval (shaded area). Statistical difference in slopes between groups was for TSH vs FT4 (A) p = 0.055, for TSH vs FT3 (B) p< 0.001, and for *G*_*D*_ vs TSH (C) p = 0.14. *G*_*D*_ values differed significantly (p<0.001) between controls and treated patients, and r^2^ for the depicted *G*_*D*_ vs LT4 dose correlation (D, p< 0.001) was 0.58. See methods section for patient characteristics.

As predicted by the model, we observed distinct alterations in both the TSH-FT4 and TSH-FT3 relationship between the group of LT4-treated patients with benign thyroid diseases (see Methods), and a control group of healthy subjects (Fig 5A and 5B). Global step-up deiodinase activity (*G*_*D*_) was positively correlated with TSH in the control group (tau 0.12, p = 0.004), but not in the treatment group (tau 0.1, p = 0.31, Fig 5C). *G*_*D*_ was inversely correlated with weight-adjusted LT4 dose in treated patients (tau -0.26, p< 0.008, Fig 5D).

**Fig 6:**
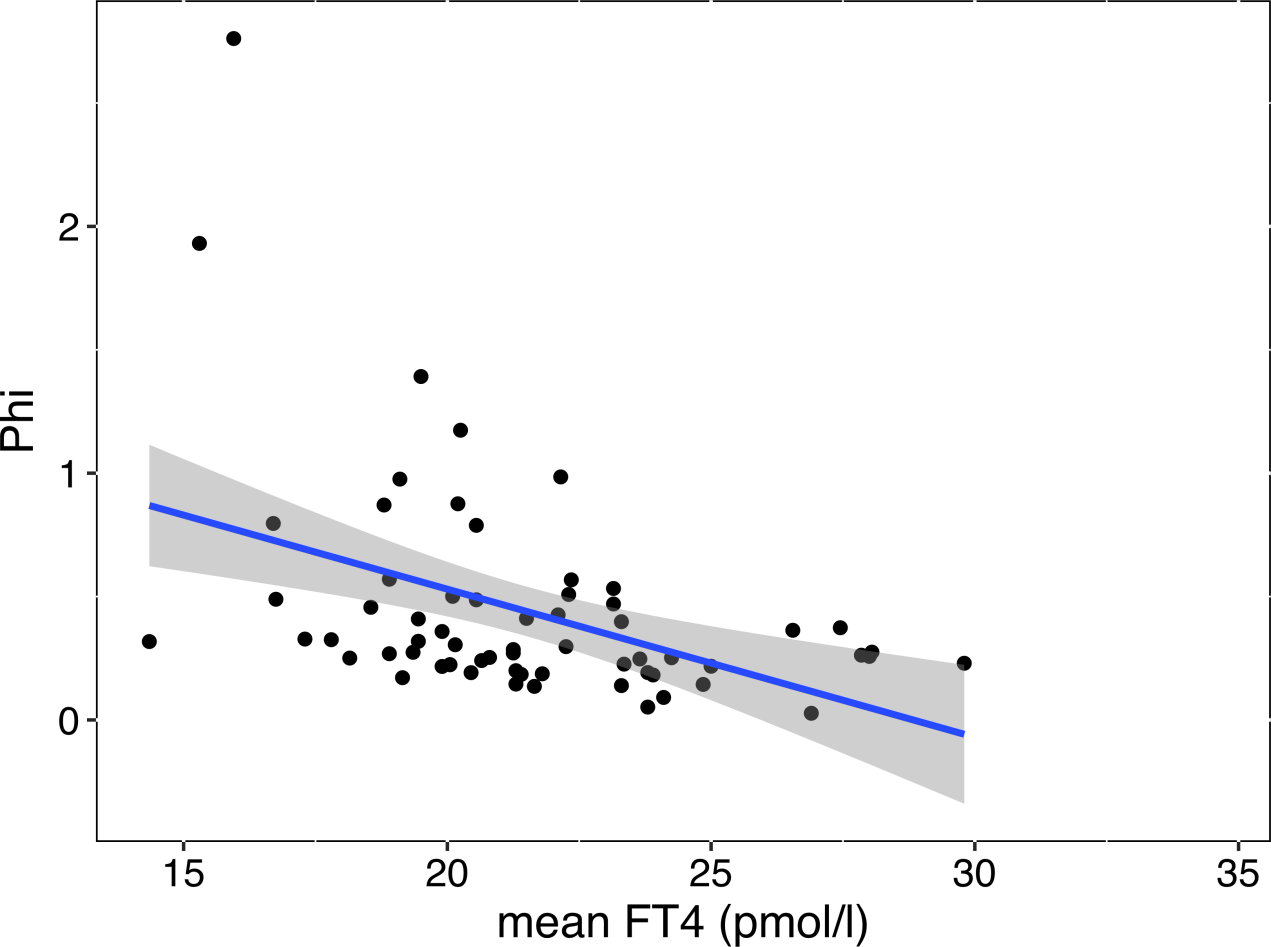
Association between phi and FT4 in LT4-treated patients with thyroid carcinoma. Phi refers to the steepness of the gradient of the TSH-FT4 relationship and was estimated as described in Methods. The robust regression line for the relationship is indicative only. The statistical significance of the relationship was based on Kendall’s tau rank correlation (see Results).

In LT4-treated patients with thyroid carcinoma followed after a dose change of at least 50 μg/day where TSH concentration remained within the range of 0.1 to 4 mIU/L, the structural parameter *phi* was estimated from the maximum and minimum concentration of corresponding TSH-FT4 pairs. It was highly correlated with thyroid function, increasing towards the hypothyroid range (Fig 6, mean FT4 tau 0.30, p<0.001, TSH tau -0.25, p = 0.004). This was still significant (p< 0.001) after correcting for dose difference.

## Discussion

The nature of the TSH-FT4 relationship in health and disease is a key factor in promoting TSH measurement as an accurate test in the diagnosis and treatment of thyroid patients [13]. The long-held tenet of a log-linear relationship between TSH and FT4 has recently been challenged by large cross-sectional studies favouring more complex models [5–10]. Conversely, others have defended the validity of the widely accepted log-linear model [22,37–41]. In an attempt to crystallise this debate by addressing the unanswered questions, we have undertaken set point simulations based on a homeostatic feedback control model. This study relates model application to the resulting practical implications. For a broader discussion of the theoretical background of homeostatic modelling and set point theory we refer to several preceding original articles and recent reviews [14,23].

To underpin our approach, we relied on a hybrid model [21] derived from earlier examples, which had been made available as open source software (see Methods). As a strength of the study, the models have been theoretically well documented and were extensively validated in a series of both older and newer studies involving more than 10,000 subjects [16,17,20,21,23–28]. For comparison of simulated with observed data points, large and clinically diverse data were accessed from a prospective cross-sectional trial [10] and retrospective long-term follow-up of patients with thyroid carcinoma [33]. Limitations in analysis emanate from the indirect approach inherent to any modelling. Also, we could only investigate partial influences of a system of much higher complexity, as reviewed elsewhere [13,42]. This limitation includes an emerging–yet still incompletely understood–supporting role of circulating FT3 in controlling pituitary TSH secretion together with the FT4 feedback, thereby among other influences shaping the TSH–FT4 relationship particularly in LT4-treated athyreotic patients [33].

The assumed uniform fixed correlation of the TSH-FT4 relationships has been widely dismissed from the results of large population studies [5–10]. However, according to some authors a log-linear relationship may still hold true for an individual person [22,23,37–41]. Attempts have been made to reconcile a log-linear intra-individual relationship with a non-log-linear population model [41,43,44]. Given inherent difficulties in clinically verifying the true nature of the relationship a modelling strategy as presented here may prove useful. When comparing theoretical models of increasing complexity, subtle but important differences arise. This demands precise specification of structural parameters and empirical testing in thyroid health and disease.

Reconstruction of individual set points can be expected to deliver a more personalised solution, compared to the population statistics that are currently used for defining the reference ranges for thyroid hormones and TSH [11,14,22,23,45,46]. We demonstrate that set points may be theoretically derived using homeostatically defined structural parameters closely resembling the paired TSH-FT4 values in a naturally observed population. Ranges are, however, difficult to compare because set points are distributed in a healthy reference population over a kite-shaped area, whereas a combination of the conventional univariate reference ranges for TSH and FT4 is bounded by a rectangle, and their bivariate representation is elliptical [12]. Divergent clinical disease classification of subclinical hypo- or hyperthyroidism may therefore arise, merely based on the use of different statistical techniques and their respective interpretation of ranges [12]. Lacking a clinical definition of subclinical disease and solely relying on laboratory ranges does, however, further contribute to a substantial dilemma when deciding on treatment requirement [3]. Given the non-normal characteristics [47] and exceptionally high individuality index (ratio of the intra-individual to inter-individual variation) of TSH [45], a validated method of set point reconstruction could aid in individualising decision thresholds and reducing diagnostic uncertainty surrounding a TSH range between 2 and 10 mIU/L [3]. For instance, in our panel of clustered set points, some patients with a TSH value of 7 mIU/L would be placed into the category of successful restoration by the regulatory elements of euthyroidism while others may have failed at this attempt. Rather than using a bivariate range thyroid reserve capacity (*G*_*T*_) may be estimated from FT4, TSH and some other constants, to provide a consolidated measure for system stress and early signal of imminent thyroid failure [47]. Apart from the regulation of glandular hormone production, which contributes only a small proportion to the T3 pool, conversion of T3 from T4 is also controlled by TSH via a feedforward motif on type 1 and type 2 deiodinases [13]. The two elements under central control, thyroid secretory capacity (*G*_*T*_) and global step-up deiodinase activity (*G*_*D*_) are, in turn, closely linked and interdependent to facilitate T3 stability [47]. This may explain the observed high inter-individual variability in both intercept and gradient of the defining TSH-FT4 relationship [33,41,48]. The central control system appears to be more dynamic than previously thought. Set point adaptation may occur in response to minor disturbance, not only dramatic events such as severe non-thyroidal illness [49]. As an example, a change in body composition in androgen deprived patients with prostatic cancer was associated with a concomitant rise in both hormones TSH and FT4, indicating a shift in regulation from the controlling to a tracking mode [49,50].

In LT4 treatment, examination of the recovery phase from hypothyroidism to euthyroidism is most relevant to define dose adequacy. While a log-linear gradient may be sufficient to approximate range-restricted segments, such as the hypothyroid region of the TSH-FT4 relationship, the relation becomes more variable and more damped within the euthyroid range [5–10,15,33]. As FT4 and TSH become increasingly uncorrelated towards the euthyroid range, influences other than TSH such as genetic factors, age and body mass index play a major role in shaping the relationship, thereby adjusting set points and fine-tuning the thyroid hormone response [5,7,9,10]. The pituitary thyroid feedback loop involves both circulating thyroid hormones, FT3 and FT4. During long-term follow-up of patients with thyroid cancer, weight adjusted LT4 dose, SPINA-GD and the resulting circulating FT3 concentrations have been shown to modulate the TSH-FT4 relationship, independently of additional confounders such as gender, age and BMI [33]. Longitudinal studies are less prone to distortions from statistical phenomena arising from a heterogeneous mix of individual set points present in a population [33,41,44–47]. Importantly, this study [33] suggested a type of cascade control. The homeostatic equilibria in LT4-treated patients have been shown to differ fundamentally from those in a state of thyroid health [10,47]. This results in substantial displacement of the appropriate ranges for FT3, FT4 and TSH, compared to the reference ranges of the hormones in the healthy population (Fig 5). Those do not equally apply to the treatment situation [10]. The displacements are clinically meaningful, as they were associated with both considerable variability in the biochemical treatment response and relief of patient symptoms [28,33,51,52].

To simulate such a scenario, we introduced moderate variation of the gradient over the functional range, technically increasing phi concomitantly with GT in the model. The pattern of set points derived by this approach resembled the TSH-FT4 pairs observed in a treatment group with benign diseases. In this comparison, patients with thyroid carcinoma were excluded, because their TSH was frequently completely suppressed and an additional role for remnant thyroid tissue has also been recognised [34,52]. Step-up deiodinase activity is diminished in the absence of remnant thyroid tissue [10,34,52], and it is inversely correlated with the LT4 dose administered (Fig 5, [33]). Conversion efficiency has been reported to decline with age [16,53], but this was not a significant confounder in our treatment group. While the gradients of the observed relationships between TSH and FT4/FT3 in LT4-treated patients was steeper, compared to controls, confirming earlier reports [10,28], the significant displacement of the ranges between the groups is also remarkable.

To further examine possible variation particularly in the gradient of the TSH-FT4 relationship, we calculated phi for each individual patient from repeated measurements of corresponding TSH-FT4 pairs. This was done in a group of patients followed long-term for cancer treatment, and within the readily measurable TSH range of 0.1 to 4.0 mIU/L following a 50 μg/day minimum change in LT4 dose. Estimates for phi were dependent on thyroid function, increasing with lower FT4 and higher TSH. In accord with our data [48,54], a large intra-individual analysis of mostly treated patients demonstrated heterogeneity in the defining relationships between TSH and FT4 and a considerable variation in the best fitting model [41]. At the pituitary level, about half of the inhibitory effect of thyroid hormones on TSH secretion has been attributed to intracellular T3 converted from circulating FT4 [55]. Sensitivity of TSH, central T3, FT3 and FT4 to changes in structural parameters has also been predicted by mathematical modelling [28], although the mathematical equations for the added T3 feedback under various conditions have not yet been worked out. On average, we estimated the relative contributions of FT4 and FT3 towards TSH suppression to be approx. 52% and 38%, respectively, while another 10% were attributable to the interaction of the two hormones [33].

Calculated parameters and relationships have emerged as novel tools in clinical research, as reviewed elsewhere [23]. The modelling approach of the TSH-FT4 relationship described here advances further in refining the process of individual set point reconstruction. The simple log-linear equation discovered many years ago is still fundamental [30], but dynamic interrelationships make the system much more adaptive than has been previously thought [13]. Although our basic understanding of the complex regulatory mechanisms involved in the hypothalamic pituitary thyroid feedback control is still rudimentary, three main elements have emerged from recent studies [10,12,16,21]; 1) a high degree of individuality with each patient having their own characteristic structural parameters that are partly genetically determined, 2) the notion of relational stability where T3 stability is maintained by an early adaption of the relationships still within the euthyroid range and 3) the conditionality of the equilibria and displacement of “normal” ranges, which are different in LT4-treated patients compared to those in healthy controls. Our simulation model of adjusted set points has addressed all three elements in order to advance the simplistic standard model, but it is still experimental at this stage.

However, disregarding the effects of altered equilibria can introduce considerable bias when interpreting TSH measurements for diagnostic use [10]. Apparently, pituitary TSH is not an adequate measure of thyroid hormone-controlled homoeostasis during LT4 treatment [28,56,57]. We suggest to replace TSH-guided dose finding with an individualised and integrated view of all three parameters FT3, FT4 and TSH and their respective equilibria. Based on set point distribution, kite-shaped reference limits for the TSH-FT4 relationship are physiologically more appropriate than a rectangular area derived by conventional univariate consideration. New treatment strategies are clinically required, as a large prospective study has recently shown that hypothyroid patients cannot expect a full symptomatic recovery from hypothyroidism with the current guideline based LT4 therapy [58].

In summary, we present a physiologically based homeostatic model that permits simulative reconstruction of individual set points. A model of high complexity incorporating both individually varying parameters for the maximum TSH response and variable gradients over the thyroid functional range provided a pattern of set points that resembled the observed data under various conditions. Applied modelling, although still incomplete and experimental at this stage, shows a potential to aid our physiological understanding of the interplay between TSH and thyroid hormones and should eventually benefit more personalised clinical decision making.

## Supporting information

S1 Table De-identified patient data in Office Open XML format. (XLSX)

S2 Table De-identified patient data in data interchange format. (DIF)

S1 File Supplementary code for the statistical platform R. (DOC)
