# Supplementary material for: Advances in applied homeostatic modelling of the relationship between thyrotropin and free thyroxine
Source: PLoS One. 2017 Nov 20;12(11):e0187232. doi: 10.1371/journal.pone.0187232 (PMC5695809; doi:10.1371/journal.pone.0187232)
Supplement: S1 File — (DOC) [file pone.0187232.s003.doc]

# Supplementary Material

## Advances in Applied Homeostatic Modelling of the Relationship Between Thyrotropin and Free Thyroxine

Rudolf Hoermann, John E M Midgley, Rolf Larisch and Johannes W Dietrich*

*** Correspondence:** Corresponding Author: johannes.dietrich@ruhr-uni-bochum.de

### Supplementary Clinical Data

De-identified clinical data used for the article are provided in a separate Excel® spreadsheet.

For more detailed information on patients and data please refer to the relevant publications that have been referenced in the article.

### Supplementary Code

Applied modelling and simulation relied on code specifically created by the authors to port the hybrid model to the statistical platform R.

R Core Team (2017). R: A language and environment for statistical computing. R Foundation for Statistical Computing, Vienna, Austria. URL <https://www.R-project.org/>. RRID: SCR_001905.

There are several requirements and considerations for the code to be executed.

1. Availability of high end computing is a requirement as the simulations demand considerable computing capacity.

The time for the code to be executed may vary considerably depending on computing power. Please be patient.

1. The R statistical base package and the required additional packages must be installed as a prerequisite for the code to run. See Methods. We used R version 3.3.1 for Mac. The current version R 3.4.2 as of this writing works as well.

Details depend on the individual set up. Please refer to the R manual for further instructions. We are assuming that any potential user is sufficiently familiar with the program and proficient in the R programming language to understand the coding sequence that is provided.

1. The R code provided below can be copied into the working console to replicate our experiment, providing R and the required helper packages are installed.

The example generates for isntance a list with 75,000 paired TSH and FT4 data points.

1. Please note that the code for the simulations involves random sampling from a uniform distribution. That means the points are expected to vary randomly, and will not look exactly the same as the ones depicted in the figure. The pattern should be similar though.

#### R code

#start with formula1 for set point reconstruction in a properly set-up R environment

setp1= function(GT, S, phi){

GT=GT

S=S

phi=phi

TSH=1

y=TSH

z=1

yy1=function(y,GT) {

GT=GT

DT=2.75

at=0.1

bt=1.1e-6

TBG=3e-7

TBPA=4.5e-6

k41=2e10

k42=2e8

x=(GT*at*y)/((bt*(DT+y)*(1+k41*TBG+k42*TBPA)))

}

yy2inv= function(y,S,phi) {

S=S

phi=phi

x= (log(y/S))/(-phi)

}

yy=function(z) yy1(z,GT) - yy2inv(z,S,phi)

ur1= multiroot(yy, 0.001)

py= ur1$root

px= yy1(ur1$root,GT=GT)

return(list(px,py))

}

setp2 <- function(GT,S,phi){

ls <- list()

for (i in 1:length(GT)) {

ls[[i]] <- setp1(GT[i],S,phi)

}

dm2 <- do.call("rbind",ls)

return(dm2)

}

setp3 <- function(GT, S.vec, phi.vec){

ls <- list()

n <- 1

for(i in 1:length(S.vec)){

for(j in 1:length(phi.vec)){

ls[[n]] <- setp2(GT, S.vec[i], phi.vec[j])

n <- n + 1;

}

}

ls <- do.call("rbind",ls);

return(ls)

}

#end of formula1

#specify the structural parameters to be sampled from a uniform distribution for modelling, where GT refers to the maximum thyroid capacity, S the intercept of the TSH-FT4 relationship and phi the gradient of the TSH-FT4 relationship.

#For more details refer to Methods, text and legends in the article as well as references for further reading.

GT.vec = seq(1.67,7.51,by=0.2)

S.vec=runif(50,100,600)

phi.vec=runif(50,0.27,0.50)

#execute the code in order to generate the set points, FT4 -TSH pairs

pts3=setp3(GT.vec,S.vec,phi.vec)

#check the dimension of the resulting list (optional)

dim(pts3)

#Plot the hypothetical points

using an xy scatterplot with x= column1= FT4 (pmol/l) and y= column2= TSH (mIU/l).

# For this purpose, the user may just use any of the many plotting options available in R they may be familiar with.

#Overlay with the clinical data.

An xy representation of the clinically observed FT4 and TSH data points may be overlaid and added to the graph of the simulated points.

Clinical data have been provided as a Supplementary File.

In case of queries or further assistance please contact the authors.
